# Real-time cognitive performance and positive symptom expression in schizophrenia

**DOI:** 10.1007/s00406-021-01296-2

**Published:** 2021-07-21

**Authors:** Maud Dupuy, Majd Abdallah, Joel Swendsen, Bernard N’Kaoua, Sandra Chanraud, Pierre Schweitzer, Melina Fatseas, Fuschia Serre, Elodie Barse, Marc Auriacombe, David Misdrahi

**Affiliations:** 1 Institut de Neurosciences Cognitives et Intégratives d’Aquitaine (INCIA), University of Bordeaux/CNRS-UMR 5287, Bordeaux, France; 2 EPHE, PSL Research University, Paris, France; 3 Handicap, Activity, Cognition, Health, Inserm/University of Bordeaux, Talence, France; 4 Addiction and Neuropsychiatry (SANPSY), University of Bordeaux, CNRS USR 3413 – Sleep, Bordeaux, France; 5 CH Charles Perrens, Bordeaux, France; 6 CHU Bordeaux, Bordeaux, France

**Keywords:** Schizophrenia, Ecological Momentary Assessment, Cognitive functions, Positive symptoms, Magnetic resonance imaging, Graph theory

## Abstract

Deficits in cognitive functions are frequent in schizophrenia and are often conceptualized as stable characteristics of this disorder. However, cognitive capacities may fluctuate over the course of a day and it is unknown if such variation may be linked to the dynamic expression of psychotic symptoms. This investigation used Ecological Momentary Assessment (EMA) to provide mobile tests of cognitive functions and positive symptoms in real time. Thirty-three individuals with schizophrenia completed five EMA assessments per day for a one-week period that included real-time assessments of cognitive performance and psychotic symptoms. A subsample of patients and 31 healthy controls also completed a functional MRI examination. Relative to each individual’s average score, moments of worsened cognitive performance on the mobile tests were associated with an increased probability of positive symptom occurrence over subsequent hours of the day (coef = 0.06, *p* < 0.05), adjusting for the presence of psychotic symptoms at the moment of mobile test administration. These prospective associations varied as a function of graph theory indices in MRI analyses. These findings demonstrate that cognitive performance is prospectively linked to psychotic symptom expression in daily life, and that underlying brain markers may be observed in the Executive Control Network. While the potential causal nature of this association remains to be investigated, our results offer promising prospects for a better understanding of the underlying mechanisms of symptom expression in schizophrenia.

## Introduction

Despite an extensive literature documenting cognitive deficits in schizophrenia, investigations to date have reported no or only a weak associations between cognitive performance and positive symptoms [1–3]. However, traditional clinical research paradigms conducted in laboratory or hospital setting are unable to accurately describe the highly dynamic interactions of psychotic symptoms and cognition as they unfold in natural contexts. Over recent decades, Ecological Momentary Assessment (EMA) has provided new insights into the mechanisms underlying symptom expression in diverse psychiatric disorders [4, 5]. For schizophrenia, these investigations have demonstrated that the occurrence of positive symptoms can be predicted by momentary changes in diverse states and behaviors, including cannabis use [6, 7], negative affect [8], autonomic nervous system arousal [9] and other constructs [10]. Despite the new insights that EMA has provided relative to processes implicated in schizophrenia, cognitive deficits has been largely ignored in investigations using this approach to date. Moreover, mobile cognitive testing has only recently been integrated into EMA research [11, 12], including for schizophrenia [13]. Such novel tools would permit the investigation of whether momentary fluctuations in cognitive performance are linked to the experience of positive symptoms in daily life.

Despite the development of multimodal neuroimaging techniques, the relationship between brain-based biomarkers of schizophrenia and symptom expression is still poorly understood. Decades of neuroimaging research have nonetheless demonstrated alterations of brain connectivity in schizophrenia, leading to the hypothesis of a “disconnectivity syndrome” [14–19]. Functional magnetic resonance imaging (fMRI) has played a prominent role in these advances, as it is the only noninvasive method for the visualization of whole-brain functional networks in humans [20]. Resting-state fMRI (rs-fMRI) measures the spontaneous brain activity based on low-frequency fluctuations in Blood Oxygen Level-Dependent (BOLD) signals during resting conditions [21], and resting-state Functional Connectivity (rs-FC) refers to the co-variation of BOLD signals between brain regions that is considered to reflect brain's intrinsic and stable functional organization [22]. A number of widely distributed resting-state brain networks show alterations in strength and architecture of functional connectivity in schizophrenia. Among them, the fronto-parietal network is known to be involved in cognitive performance, and particularly in executive functioning [23, 24], that may play an important role in the pathophysiology of this disorder. Recently, mathematical Theory of graphs has emerged as a powerful framework to investigate intrinsic brain networks overall organization or topology based on rs-fMRI data [25, 26]. This framework defines the brain as a graph consisting of nodes (brain regions) linked by edges (functional inter-connections). Graph theory enables investigation of brain properties that are not detectable by studies of unique brain regions or pairwise connections between Regions of Interest (ROIs). Through the calculation of mathematical indices called “metrics”, graph theory makes it possible to evaluate properties of brain networks that reflect their capacity to transmit information. Some of these metrics are related to the concept of functional segregation (i.e. the existence of functionally specialized regions) and others to the concept of functional integration (i.e. the fact that these specialized regions are organized in interacting functional networks). These network properties have been found to be consistently altered in schizophrenia [20, 26–36]. The combination of graph theory analyses with EMA data concerning the dynamic within-person association of cognitive fluctuations and symptom expression would provide valuable insight into the complex brain-daily life experience in schizophrenia.

The present controlled investigation examines the role of momentary fluctuations in cognitive performance and the experience of positive symptoms in patients with schizophrenia. For a one-week period, participants provided symptom-related information via EMA as well as completed mobile tests of objective cognitive performance. A subsample of participants was also administered fMRI before commencing the EMA phase of the investigation. The objectives of this study were: (i) characterize the within-person association of fluctuations in cognitive performance with the occurrence of positive symptoms over subsequent hours of the day; (ii) integrate EMA data into graph-based, resting-state network MRI analyses to describe functional brain mechanisms linked with these associations. We hypothesized that a momentary decrease in cognitive performance would be associated with the expression of positive symptoms in daily life and that this relationship would also be associated with metrics of the fronto-parietal executive control network.

## Methods

### Participants

Seventy-five individuals were recruited for the present investigation (33 patients with schizophrenia and 42 healthy controls) in the context of regular consultation at a university hospital in southwest France. All participants provided written informed consent and the study was approved by the national ethics review committee. The Mini International Neuropsychiatric Interview French Version 5.0.0 (MINI, Sheehan et al. [37]) was used to confirm or rule out DSM-IV-TR diagnoses. Patients met criteria for schizophrenia, were receiving antipsychotic medications and were evaluated as clinically stable outpatients by a staff psychiatrist. Healthy control participants were identified through community postings and were recruited in the absence of lifetime psychotic disorder, lifetime bipolar disorder, and lifetime substance dependence, as well as no other current DSM-IV-TR axis I disorder. All participants were also required to be free from conditions or disability incompatible with the use of a smartphone or any contraindication for an MRI examination.

### Procedures

After verification of eligibility criteria, participants completed a clinical and neuropsychological assessment battery (see below for details) and were then trained to operate a study-dedicated smartphone (Samsung Galaxy S with a 10.6 cm screen, 12-point font size). Following successful completion of this training, they were given a smartphone to carry with them for 1 week and were instructed to respond to five electronic surveys per day. The surveys occurred at random intervals within 5 equal time epochs from morning to evening (approximately every 3 h). A mobile color-word interference test of cognitive performance that was similar to the Stroop test was administered at the end of two of the five daily surveys (for a more detailed description, see below). The average duration of electronic surveys was approximately 5 min. The subsample of individuals who also received an MRI examination did so rapidly (approx. 48 h) before completing clinical testing and EMA. Participants received compensation up to 100 euros in purchase vouchers for the completion of both the EMA and MRI phases of the study.

### Clinical and neuropsychological assessment battery

#### Mini International Neuropsychiatric Interview French version 5.0.0 [37]

The MINI is a brief structured diagnostic interview (median: 15 min), exploring the main clinical disorders of DSM-IV, including substance dependence comorbidity.

#### Positive and negative syndrome scale [38]

French version (Lépine et al. 1989). This scale is a hetero-evaluation scale of psychopathological symptoms observed in patients with psychotic syndromes, especially individuals with schizophrenia, based on 30 items ranging from 1 (absent) to 7 (extreme). It allows to calculate the scores of three dimensions: positive symptoms (7 items), negative symptoms (7 items) and general psychopathology (16 items). This scale is administered to patients only.

#### Stroop test

The Stroop paradigm is a common procedure for measuring executive functions, notably cognitive inhibition. The test is composed of three parts: a word page (color words printed in black ink), a color page (color hues printed in rectangle) and a competing word-color page (e.g., the word ‘red’ printed in blue ink). Each page contains five columns of ten items. Participants were instructed to read the maximum number of words (word page) and name the maximum ink colors as quickly as possible, in 45 s.

### EMA surveys and mobile cognitive tests

The EMA surveys included questions about physical location, activity, and mood states at the moment of survey completion, as well as concerning the experience of positive psychotic symptoms since the previous EMA survey. Psychotic symptoms were those validated by previous EMA investigations [7, 39] and included five questions concerning delusions of being spied on, mind reading, thought insertion, thought broadcasting, and having special powers. An additional question assessed the experience of visual or auditory hallucinations. Psychotic symptoms were assessed within each of the five daily electronic questionnaires. The presence of any psychotic symptom at each electronic assessment was defined as endorsement of at least one of these six questions. In other words, the presence or absence of at least one of the psychotic symptoms (as a binary variable) was the main outcome of the behavioral experiment. The mobile test of cognitive performance was similar to the interference trial of the Stroop Test and provided participants with a list of 16 color words (four lines each of “Yellow”, “Red”, “Blue”, and “Green”) and in different colors (also Yellow, Red, Blue, Green). No word was written in the color that matched the meaning of the written word (i.e., the word “Blue” would appear in the colors of red, yellow, and green, but not in blue). The order of words and colors was randomized, and each word and color appeared once per line. Participants were instructed to say the ink color of each word aloud as quickly as possible. Participants were provided a maximum of 60 s to complete the task. When the participants completed this naming, they were instructed to press the ‘finished’ button displayed on the screen that would stop the timing of test. The mobile color-word interference test was presented at the end of two of the five daily electronic questionnaires (providing a maximum of 14 distinct mobile test scores). To avoid biases associated with test repetition or time of the day, fourteen unique versions of the test were administered during the week in an order that was counterbalanced across the different time epochs of the day. Responses were audio recorded on the study smartphones, and each audio file was imported into Audacity® software to determine the precise time needed to complete the task, in seconds. This duration corresponds to the time elapsed between the first word spoken and the last word spoken in relation to the task. Each audio file was listened to and scored independently by two trained raters. Inter-rater reliability for this coding procedure was above *r* = 0.90. As this test resulted in relatively few errors, the time to complete the mobile color-word interference test was used as the primary measure of cognitive performance.

Based on the present sample, an initial publication examined compliance rates, fatigue effects, training effects and convergent validity [13]. Compliance with the self-report EMA interviews was high for all participants, with 95% of the possible assessments being completed by both controls and patients in the context of their daily lives (resulting in 1654 observations). Examination of compliance with mobile cognitive tests revealed that 88% and 83% of the mobile letter-word generation assessments were completed by controls and patients, respectively. No fatigue effects were observed considering that there was no variation in the number of missing observations occurred as a function of day of the study. Concerning practice effects, defined as the time needed to complete the color-word interference test, a significant decrease was observed as a function of study duration for both groups. Finally, analyses found significant correlations between the mobile test and the traditional Stroop test for both groups, indicating that both measure the same underlying construct.

### MRI examination

Brain imaging data were collected using a 3.0 Tesla GE MRI system with a 32-Channel MRI Head Coil. Anatomical MRI volumes were acquired using a sagittal three-dimensional T1-weighted (Repetition Time = 8.5 ms, Echo Time = 3.2 ms, flip angle = 11°, FOV = 256 mm × 256 mm, voxel size = 1 mm × 1 mm × 1 mm, Slice Thickness = 1 mm, 176 slices). The resting-state functional images were collected using a single-shot echo-planar sequence (RT = 2.2 s, ET = 27 ms, flip angle = 80°, FOV = 192 mm × 192 mm, voxel size = 3 mm × 3 mm × 3.5 mm, 42 axial slices). For the resting-state scan, participants were instructed to keep their eyes closed, to not fall asleep and to not think about anything in particular.

### Statistical analyses

#### EMA data

**Fig. 1:**
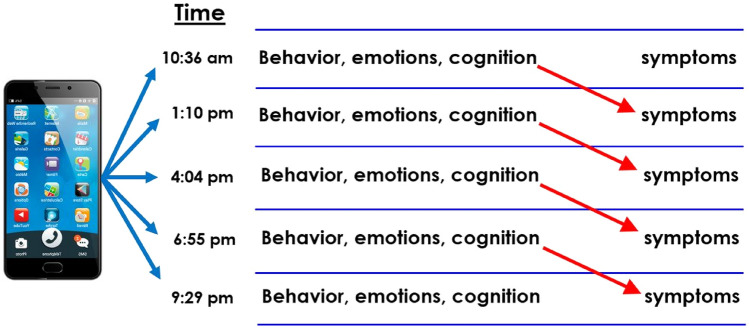
EMA cognitive testing in the prediction of positive symptoms

Analyses of predicting the occurrence of positive symptoms were conducted only on the sample of patients with schizophrenia. Prospective within-day associations between cognitive performance and psychotic symptoms were analyzed using hierarchical linear and nonlinear modeling [40]. Data were time-lagged so that time to complete the color-word interference test at any given assessment (T0) predicted the presence of psychotic symptoms at the subsequent assessment on the same day (T1). All analyses adjusted for the status of the T outcome variable as measured at the T0 assessment. Bernoulli models were used for dichotomous outcomes (presence or absence of psychotic symptoms). Multiple or exploratory analyses were avoided in light of the a priori hypothesis concerning the impact of cognitive performance on the subsequent occurrence of psychotic symptoms. Figure 1 presents an illustration of the EMA methodology, where red arrows indicate the prospective, within-day association between momentary cognitive performance and later-occurring symptoms. These daily coefficients (or arrows) are then aggregated across days for each individual before being averaged for the sample as a whole.

#### fMRI data pre-processing and graph analysis

Neuroimaging data in this investigation were based on pre-processing performed using the latest version of FMRIPREP [41], a Nipype-based tool. For each subject, the T1-weighted (T1w) volume was corrected for intensity non-uniformity (INU) using ANTs N4BiasFieldCorrection v2.1.0 [42] and skull-stripped using ANTs antsBrainExtraction v2.1.0 (using the OASIS template). Spatial normalization to the ICBM 152 Nonlinear Asymmetrical template version 2009c [43] was performed through nonlinear registration with the antsRegistration tool of ANTs v2.1.0 [44], using brain-extracted versions of both T1w volume and template. Brain tissue segmentation of cerebrospinal fluid (CSF), white-matter (WM) and gray-matter (GM) was performed on the brain-extracted T1w using fast^50^ FSL v5.0 [45]. Functional data were slice time-corrected using 3dTshift from AFNI v16.2.07 [46] and motion-corrected using mcflirt (FSL v5.0.9, [47]). Distortion correction was performed using an implementation of the TOPUP technique [48] using 3dQwarp (AFNI v16.2.07 [46]). This was followed by co-registration to the corresponding T1w using boundary-based registration [49] with nine degrees of freedom, using flirt (FSL). Motion correcting transformations, field distortion correcting warp, BOLD-to-T1w transformation and T1w-to-template (MNI) warp were concatenated and applied in a single step using antsApplyTransforms (ANTs v2.1.0). Six head-motion parameters along with WM and CSF mean signals were used as noise regressors within a GLM framework. In addition, ICA-based Automatic Removal Of Motion Artifacts (AROMA) was used to generate aggressive noise regressors as well as to create a variant of data that is non-aggressively de-noised [50]. Spatial smoothing was avoided following [51], and bandpass filtering (0.008–0.1 Hz) was performed. Many internal operations of FMRIPREP use Nilearn [52], principally within the BOLD-processing workflow.

**Fig. 2:**
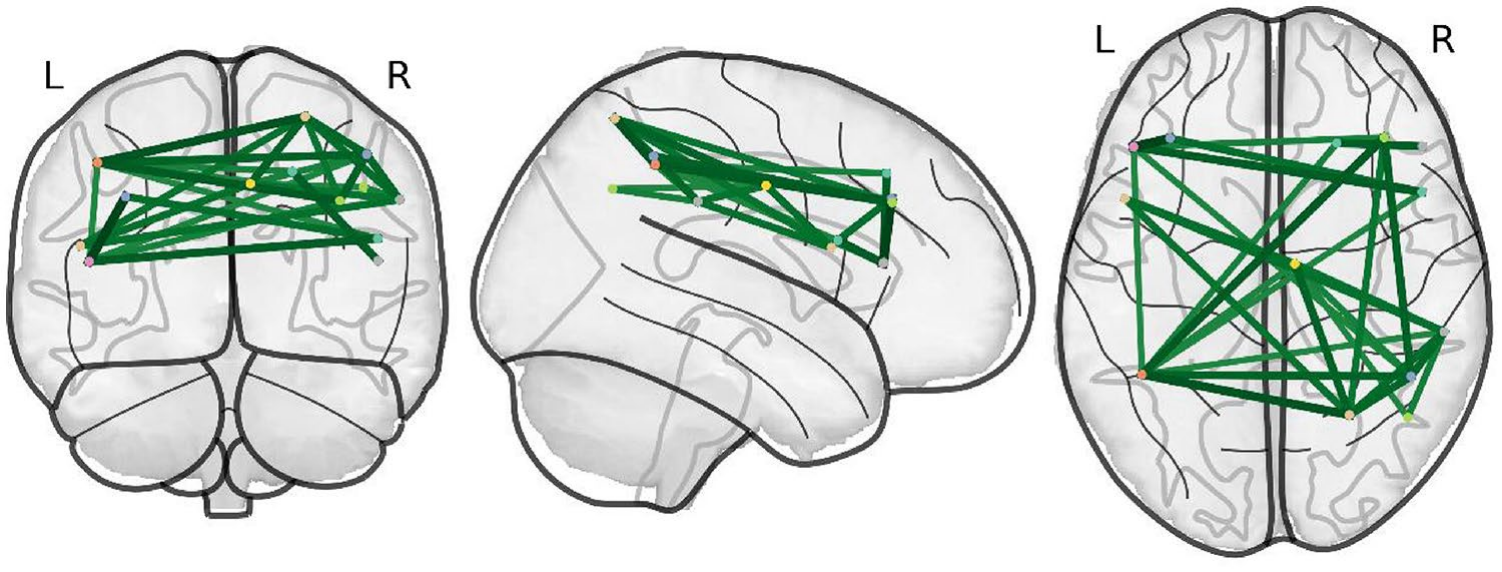
Illustration of graph theory as applied to the Executive Control Network in an example patient with schizophrenia

Graph analysis was performed using PyNets (https://github.com/dPys/PyNets). First, for each participant, BOLD signals were extracted from 13 ROIs included in the AAL2 atlas and that fall under an Executive Control Network (ECN) mask created from the intrinsic connectivity 7-Network Yeo atlas [53]. To illustrate the application of graph theory to the Executive Control Network, the data for one example participant were reconstructed by PyNets and are provided in Fig. 2. The Pearson correlation between pairwise ROIs was calculated to create the correlation adjacency matrix for each participant. A proportional threshold strategy was performed to prune negative and weak connections that might be spurious and preserve a percentage of the strongest positive connections [54]. A range of proportional thresholds was used based on previous literature [0.35–0.5] (i.e. the top [35–50%] of the edges of the graph survive thresholding). Binarization was not performed and weighted graph metrics were calculated. Several well-known graph metrics were computed to characterize the participant-level ECN: Global Efficiency, Average Local Efficiency, Small-worldness, Degree Assortativity Coefficient, Average Clustering, Average Shortest Path Length, Graph Number of Cliques, Transitivity, Modularity, and Coreness [55]. The different metrics are calculated from elementary properties of the graphs, including degree (the number of links of a given node), path (the sequence of edges connecting a set of successive nodes) and length (the number of edges constituting the path).

Finally, a two-level statistical model was then constructed using hierarchical linear and nonlinear modeling to explore the correlations between average prospective relationships of cognition and positive symptoms during EMA period and graph theory metrics of the Executive Control Network. The first level included variability in time-lagged EMA ratings of cognitive performance (time, measured in seconds, to complete a color-word interference test) and its association with later positive symptoms, nested within persons. The second level included between-person variability in the person-level index of connectivity (graph theory indexes: Global Efficiency, Average Local Efficiency, Smallworldness, Degree Assortativity Coefficient, Average Clustering, Average Shortest Path Length, Graph Number Of Cliques, Transitivity, Modularity, Coreness). This two-level model indicated association between average prospective relationships of cognition and positive symptoms during EMA period and an index of connectivity. In other words, this model would identify cross-level interactions, which would indicate that the within-person and prospective associations between cognitive performance and symptoms varied as a function of individual difference in graph theory indices.

## Results

### Sample description

The sociodemographic, clinical, neuropsychological and mobile assessment characteristics of the 33 patients who completed the EMA phase of the study are presented in Table 1, along with those of healthy controls to aid interpretation of the clinical findings. Compared to patients with schizophrenia, healthy controls were more likely to be women, to have more years of education, and to be employed. A Mann–Whitney *U* test was conducted to determine if there were differences in response time and number of errors between patients with schizophrenia and healthy controls. Distributions of the response time and number of errors for patients and control subjects were dissimilar. Response time for patients (mean rank = 44.5) were significantly higher than for controls (mean rank = 32.9), *U* = 263, *z* =  − 4.5, *p* < 0.001, as was the number of errors, *U* = 263, *z* =  − 4.5, *p* < 0.05 (mean rank = 51.0, mean rank = 27.76, for patients and control subjects, respectively). A Spearman’s rank-order correlation was used to assess the relationship between response time and number of errors in patients within schizophrenia group. There was a strong positive correlation between response time and number of errors, rs (33) = 0.401, *p* < 0.05. These results suggest that the higher the response time to the word-color interference task, the lower the performance. A subsample of 13 patients with schizophrenia also participated in the functional MRI phase of the study.

**Table 1:** Demographic, clinical, neuropsychological and mobile assessment characteristics of the sample

	Patients (*N* = 33)	Healthy controls (*N* = 42)	*p*
Demographics			
Men, n (%)	24 (73%)	21 (50%)	*0.046*
Age	33.9 ± 10.0	33.1 ± 8.4	0.769
Level of education (years)	12.1 ± 2.1	14.5 ± 2.9	*0.000*
Employment status, (*n* and % unemployed)	4 (12.1%)	40 (95.2%)	*0.000*
Clinical characteristics			
Age at first psychotic episode (*N* = 28)	23.6 ± 6.2	**–**	
Duration of illness (*N* = 28)	9.54 ± 8.2	**–**	
PANSS positive symptoms	15.9 ± 4.0	**–**	
PANSS negative symptoms	17.7 ± 6.1	**–**	
PANSS general psychopathology	35.4 ± 7.1	**–**	
PANSS total	69.0 ± 14.7	**–**	
Positive subtype, *n* (%)	5 (15.2%)	**–**	
Negative subtype, *n* (%)	9 (27.3%)	**–**	
Mixed subtype, *n* (%)	0	**–**	
Not specified, *n* (%)	19 (57.6%)	**–**	
Substance comorbidity, *n* (%)	20 (61%)	**–**	
Neuropsychological assessments			
Stroop raw word score	92.0 ± 18.8	109.2 ± 16.6	*0.000*
Stroop raw color score	74.9 ± 13.4	96.7 ± 15.1	*0.000*
Stroop raw color-word score	47.2 ± 12.0	64.7 ± 15.7	*0.000*
Mobile cognitive test scores			
Color-word interference task response time (s)	14.66 ± 39.9	10.6 ± 23.6	*0.000*
Color-word interference task error	0.51 ± 1.8	0.08 ± 0.2	*0.016*

### Prospective association of mobile cognitive performance and later symptoms

**Fig. 3:**
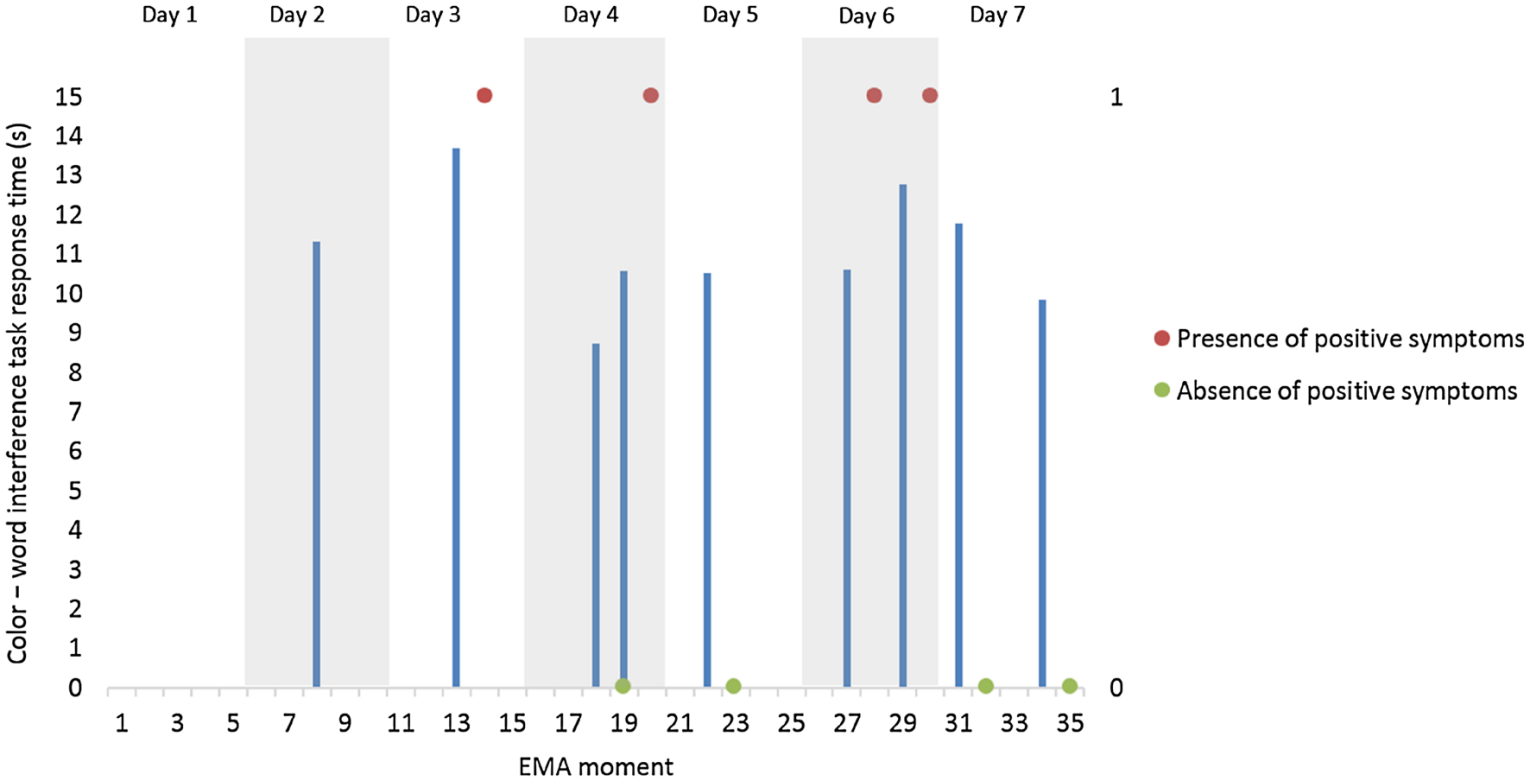
Example of the prospective within-day association between cognitive performance and positive symptoms in one patient with schizophrenia. Figure 3 is a graphical reproduction of the actual data collected for one patient in the sample. It shows on the *x*-axis the 35 moments of ecological evaluation (5 per day for 7 days). Psychotic symptoms are addressed at each of the 5 daily electronic questionnaires. A red circle represents the presence of any positive symptom and a green circle represents the absence of any positive symptom as experienced by this participant. The mobile color-word interference cognitive test was presented at the end of two of the five daily electronic interviews. The time, in seconds, to perform each completed interference test is represented by blue bars

In individuals with schizophrenia, time to complete the color-word test was associated with subsequent increases in the risk of psychotic symptoms (Coefficient = 0.06, SE = 0.02, *p* < 0.05, Odds Ratio = 1.07), adjusting for the T0 status of T1 outcome variable. Additional analyses to control for potential confounding variables confirmed that this prospective association was independent of age, gender, and time of day. However, the strength of this association increased in the presence of comorbid substance use disorders (Coefficient = 0.10, SE = 0.04, Odds Ratio = 1.10). Figure 3 illustrates the prospective within-day association between executive performance and positive symptoms for one example patient with schizophrenia who responded to 9 (64%) of the mobile cognitive tests.

### fMRI markers associated with cognitive performance and symptom expression

The significant prospective association between mobile cognitive performance and the occurrence of psychotic symptoms was itself associated with graph theory metrics of Executive Control Network (Table 2). In particular, these results indicate that the intensity of the relationship between response time and positive symptoms depends on the levels of Smallworldness, Transitivity and Graph Number of Cliques of the Executive Control Network. The Smallworldness index describes a functional organization composed of specialized modules that are globally interconnected. This type of network functioning is based on an optimal balance between integration and functional segregation and therefore describes optimal network functioning. Transitivity refers to the probability that the network has interconnected adjacent nodes, indicating the presence of closely connected clusters, or subset of regions. The number of cliques is the number of subsets of nodes in the network where the nodes are all connected to each other by edges. Both Transitivity and Number of cliques relate to the concept of functional segregation, i.e. the existence of functionally specialized regions, and describe the modular functional architecture of a graph from the organization of its nodes.

**Table 2:** Association between cognitive performance/symptom slope and graph theory metrics of the Executive Control Network

Assessment variable	Coefficient	SE	Odds ratio
Level-1			
Average slope of executive performance and positive symptoms	0.23*	0.09	1.26
Level-2			
Smallworldness	0.20*	0.06	1.22
Transitivity	0.81*	0.23	2.25
Graph number of cliques	− 0.02*	0.01	0.98

*p < 0.05, adjusting for T0 status of T1 outcome variable. Note: HLM cannot estimate the model with age and sex specified as level-2 covariates

As a function of increasing values of Smallworldness (Coefficient = 0.20, SE = 0.06, Odds Ratio = 1.22) and Transitivity (Coefficient = 0.81, SE = 0.23, Odds Ratio = 2.25), the slope of the association between poor cognitive performance and the probability of symptom became stronger. By contrast, this slope became weaker as a function of the Number of Cliques (Coefficient =  − 0.02, SE = 0.01, Odds Ratio = 0.98). An additional analysis with independent-samples *t*-test was then conducted to determine if there were differences in Smallworldness, Transitivity and Number of Cliques and between patients and controls (not shown). Network graph measures for each group were normally distributed, as assessed by Kolmogorov–Smirnov test (*p* > 0.05). These Executive Control Network metrics for patients and controls were not statistically significantly different.

## Discussion

The principal finding of this study is that momentary decreases in cognitive performance predict the occurrence of positive psychotic symptoms over subsequent hours of the day. This prospective association provides novel evidence for the association of cognitive functioning and symptom expression in schizophrenia, and it is in contrast with cross-sectional studies reporting that positive symptoms are unrelated or only weakly associated with neurocognitive performance [1–3]. The present findings therefore suggest that investigations of global capacities or static characterizations of cognitive deficits may ignore the crucial role that dynamic variation could play in the real-time occurrence symptoms.

At least three distinct hypotheses may explain this association, including the possibility that alterations in cognitive performance may constitute a “micro” prodromal state that precedes (but is not causally implicated in) symptom expression. As such, cognitive dysfunction may constitute a marker of risk that is observable early in the period of symptom occurrence or exacerbation. A second possibility is that momentary cognitive dysfunction is associated with a third variable that is itself directly associated with the probability of symptom expression. In fact, a large diversity of other variables assessed by EMA have been linked in real-time to positive symptoms, including a range of physical, emotional and behavioral states [6, 8–10]. Due to their inter-correlations, tests of the distinct roles of specific variables in future investigations would require multivariate analyses of numerous candidate predictors. Finally, rather than constituting a simple correlate of other predictors, it is possible that cognitive performance fluctuations play a direct or indirect role in the occurrence of positive symptoms. Should this final hypothesis be confirmed, it would suggest that mobile cognitive testing may provide opportunities for new, real-time therapeutic interventions.

A subsample of participants completing the EMA surveys also completed functional neuroimaging and data obtained were analyzed using graph theory. This framework defines the brain as a graph consisting of nodes (i.e. brain regions) linked by edges (i.e. functional inter-connections). Graph theory-based analyses of rs-fMRI data help to identify how the brain’s intrinsic networks are organized, and it can provide lacking information about clinical manifestations and intermediate phenotypes in schizophrenia [32, 56]. The present analyses indicate that the within-day prospective associations between cognitive performance and positive symptoms depend on the local and “small-word” organization of the Executive Control network. Otherwise stated, the relationship between cognition and symptoms is associated with functional organization of the brain in individuals affected by schizophrenia. These findings provide new insight into the nature of cognitive impairment in this disorder and its underlying brain correlates. In schizophrenia, it has been shown that altered functional connectivity reflects a stable brain function characteristic that is present across cognitive states and contexts [23, 30]. It is therefore not surprising that an association of cognitive performances, their variation and the expression of positive symptoms may be based on long-term functional characteristics of the brain. An interesting result concerns the reduction in the number of cliques as a function of the strength of the association of cognitive performance and positive symptoms. The number of cliques indicates the robustness of communication and the core-periphery organization in the network. This suggests that the better the communication is within the network, the less intense is the detrimental relationship between cognitive performance and symptoms. This result is consistent with previous studies that have demonstrated an increase in network robustness in schizophrenia [27, 32]. This increased robustness has been proposed to represent a potential functional benefit or compensation within an array of dysfunctions and deficits [30].

One limitation of the present study is its reduced sample size for neuroimaging analyses. A power analysis was conducted for the larger study from which this sub-sample is derived (with four groups of patients having different psychiatric conditions), but it was not used to determine the size of the present sample. For this reason, the present study did not examine all potentially relevant networks but focused on the executive control network as the most pertinent candidate for neuroimaging analyses. The findings should therefore be considered as preliminary evidence for a significant relationship between these EMA and neuroimaging variables, and future studies should verify these results using larger sample sizes and multiple fMRI sessions. Furthermore, the effect size of the main behavioral finding is also small. Nevertheless, this is the first study of its kind and pursuit of this topic may have important clinical implications should the predictive role of momentary cognitive performance relative to psychotic symptoms be confirmed. It should also be noted that a small effect size was observed when using only one mobile test, and therefore that other (and stronger) associations may be observed when applying a larger battery of mobile assessments. The EMA component of the investigation was also focused exclusively on the time required to complete the mobile color-word interference test, and it cannot confirm the specificity of the findings relative to other cognitive capacities. Another consideration is relative to the mobile color-word interference test used in this study. Although it was modeled after one of the most frequent neuropsychological tests of executive capacities, the development of other mobile tests would further our understanding of the precise nature or components of executive performance that are associated with positive symptom expression. Furthermore, symptom data were not collected from control participants but there is increasing evidence that psychotic-like symptoms occur at considerable frequency in healthy samples [57–59]. Attention to the full spectrum of cognitive functions and psychotic symptoms may reveal particularly novel associations in future studies.

## Conclusion

To our knowledge, this is the first investigation to examine the real-time association of cognitive performance and positive symptoms in patients with schizophrenia. The novel combination of EMA data with that of graph-based resting-state MRI networks also provides insight into the possible brain pathophysiology underlying this relationship. The current findings nonetheless constitute a first step towards better understanding dynamic cognitive performance in schizophrenia and its correlates in brain functioning.
